# Spinal anesthesia for lumbar spine surgery correlates with fewer total medications and less frequent use of vasoactive agents: A single center experience

**DOI:** 10.1371/journal.pone.0217939

**Published:** 2019-06-13

**Authors:** Hao Deng, Jean-Valery Coumans, Richard Anderson, Timothy T. Houle, Robert A. Peterfreund

**Affiliations:** 1 Department of Anesthesia, Critical Care and Pain Medicine, Massachusetts General Hospital, Boston, Massachusetts, United States of America; 2 Department of Neurosurgery, Massachusetts General Hospital, Massachusetts General Hospital, Boston, Massachusetts, United States of America; Mayo Clinic, UNITED STATES

## Abstract

**Study objective:**

Anesthesiologists at our hospital commonly administer spinal anesthesia for routine lumbar spine surgeries. Anecdotal impressions suggested that patients received fewer anesthesia–administered intravenous medications, including vasopressors, during spinal versus general anesthesia. We hypothesized that data review would confirm these impressions. The objective was to test this hypothesis by comparing specific elements of spinal versus general anesthesia for 1–2 level open lumbar spine procedures.

**Design:**

Retrospective single institutional study.

**Setting:**

Academic medical center, operating rooms.

**Patients:**

Consecutive patients (144 spinal and 619 general anesthesia) identified by automatic structured query of our electronic anesthesia record undergoing lumbar decompression, foraminotomy or microdiscectomy by one surgeon under general or spinal anesthesia.

**Interventions:**

Spinal or general anesthesia.

**Measurements:**

Numbers of medications administered during the case.

**Main results:**

Anesthesiologists administered in the operating room a total of 10 ± 2 intravenous medications for general anesthetics and 5 ± 2 medications for spinal anesthetics (-5, 95% CI -5 to -4, p<0.001, univariate analysis). Multivariable analysis supported this finding (spinal versus general anesthesia: -4, 95% CI -5 to -4, p<0.001). Spinal anesthesia patients were less likely to receive ephedrine, or phenylephrine (by bolus or by infusion) (all p<0.001, Chi-squared test). Spinal anesthesia patients were also less likely to receive labetolol or esmolol (both p = 0.002, Fishers’ Exact test). No neurologic injuries were attributed to, or masked by, spinal anesthesia. Three spinal anesthetics failed.

**Conclusions:**

For routine lumbar surgery in our cohort, spinal compared to general anesthesia was associated with significantly fewer drugs administered during a case and less frequent use of vasoactive agents. Safety implications include greater hemodynamic stability with spinal anesthesia along with reduced risks for medication error and transmission of pathogens associated with medication administration.

## Introduction

Surgical procedures on the lumbar spine include discectomy, foraminotomy, synovial cyst removal, decompression, and several types of fusions. Patients typically receive general anesthesia (GA) for these procedures. However, several primary clinical research publications describe administering spinal anesthesia (SA) for lumbar spine surgery [1–21]. Recent review articles [22–25] summarize the data from these primary reports. Some of the published studies contain comparisons between GA and SA for various hemodynamic parameters.

Sympathectomy caused by SA can cause decreased blood pressure, requiring pharmacologic intervention. A few reports identify the vasoactive drugs (atropine and/or ephedrine) used to manage hemodynamics during SA for lumbar spine surgery [2, 8–10, 26, 27]. Other publications state that vasoactive agents were used, but they are not identified [6, 7, 12, 17, 21]. A number of studies do not report whether vasoactive drugs were used to manage hemodynamics [4, 5, 11, 13, 14, 16, 18]. Finsterwald et al [21] define hypotension as an interval lasting more than one minute with systolic blood pressure less than 20% from the pre-induction baseline. They report a highly significant difference between GA and SA for the number of hypotensive episodes and for the difference in the number of patients receiving vasoactive agents to support blood pressure. These investigators do not specify which vasoactive agents were used. Only one study compares SA with GA for the percentage of cases where a specific vasoactive agent, ephedrine, was administered [2]. The investigators report giving ephedrine in 22.9% of GA cases and 36.1% of SA cases, stating that the difference was not significant. To counter blood pressure elevations associated with GA for lumbar spine surgery, a few publications [2, 5–8, 11] mention deepening the anesthetic. Only one trial describes the use of a specific agent, nitroglycerin, to treat blood pressure elevations [8]. Furthermore, it is unclear whether descriptions of hemodynamics reflect whether interventions with vasoactive agents provided the reported stability.

Anesthesiologists at our hospital commonly administer SA for routine lumbar surgery including simple decompression, microdiscectomy, and foraminotomy. Anecdotal impressions suggested that SA works well for these procedures. Compared to GA, SA appeared to be associated with less frequent use of agents to manage blood pressure. Also compared to GA, SA appeared to be associated with a smaller number of intravenous medications administered in the operating room by the anesthesiologist. We hypothesized that reviewing our data would confirm these impressions. To test this hypothesis we designed this retrospective, single-institutional study comparing specific elements of SA versus GA for 1–2 level lumbar spine procedures. One essential feature of the data set is that SA and GA case management was at the assigned anesthesia team’s discretion; our institution has no protocols. A second key feature of the data set is that over 100 different attending anesthesiologists supervised or personally administered the anesthetics. These features suggest that the analysis results would reflect “real world” practice where individual anesthesiologists manage their cases centered on the unique clinical needs of a particular patient.

## Materials and methods

The Partners Healthcare Institutional Review Board approved this study (Number: 2016P002684). Informed consent was not required due to the retrospective cohort nature of study design.

### Patient population

This study extended our previous report [28]. The patient cohort included all patients identified by structured query of our Metavision electronic anesthesia record (IMDsoft, Dedham, Massachusetts USA) undergoing lumbar microdiscectomy for herniation, lumbar foraminotomy for radiculopathy or lumbar decompression for stenosis completed by a single surgeon (JVC) at a single institution between October 1, 2008 and April 1, 2016. Data collection was terminated due to a change in the electronic anesthesia records platform.

### Inclusion / Exclusion criteria

Patients were offered SA or GA based initially on the joint discretion of the attending anesthesiologist and surgeon, followed by a consent discussion with the patient who had the right to refuse the option of SA. Spinal anesthesia was considered contraindicated for procedures planned to exceed more than 2 lumbar spinal levels and also for patients whose preoperative imaging suggested significant stenosis likely to impede rostral spread of the spinal anesthesia drug, e.g. at L1-L2, (See S1 Fig).

Spinal anesthetics were not offered to patients with known or predicted difficult airways or significantly reduced cervical spine mobility because the backup plan for inadequate SA was Laryngeal Mask Airway (LMA) insertion in the prone position. Other contraindications included extreme anxiety, a body habitus felt to be unfavorable for respiratory comfort while in prone position, and significant valvular heart disease (i.e. severe aortic stenosis).

### Conduct of anesthesia

The specifics of each anesthetic were determined by the anesthesiologist assigned to the case. No protocols guided anesthetic management of an individual patient. For GA cases, 115 individual members of the attending staff anesthesiologists either supervised or directly provided general endotracheal anesthesia; no anesthesia providers were excluded from the analysis. For the SA cases, 38 individual members of the attending staff anesthesiologists either supervised or directly provided anesthesia care; no anesthesia providers were excluded from the analysis.

SA was typically administered with the patient in sitting position on the transport stretcher, monitored with ECG, non-invasive blood pressure and pulse oximetry. Patients received intravenous sedation (typically midazolam and/or fentanyl) for spinal needle insertion (25 gauge Whitacre, 24 gauge Sprotte or 22 gauge Quincke) at the discretion of the anesthesia team. Further intraoperative sedation, e.g. propofol infusion, midazolam or fentanyl, was administered at the discretion of the anesthesia team or by patient request. During some cases the patient received the antiemetic ondansetron. Other agents occasionally employed included hydromorphone, morphine and dexmedetomidine.

GA was typically administered with propofol as the induction agent, a muscle relaxant to facilitate laryngoscopy and endotracheal intubation, an opioid for analgesia, and either inhalation or intravenous anesthetics for maintenance. Agent selection and doses were at the discretion of the anesthesia team based on clinical judgment of individual patient needs.

For both SA and GA, hemodynamic management was individualized based on the anesthesia team’s clinical judgment of patient needs. Choices and doses of vasoactive agents were at the discretion of the team.

### Description of the surgical procedure

For SA patients, after instillation of the spinal anesthetic, the patient was placed in the supine position on the transport stretcher followed by insertion of a urinary catheter. A sensory examination was performed by pinprick test and/or temperature discrimination with an alcohol swab. After confirming that a satisfactory level had been achieved (typically at the level of the T6 –T8 dermatome), the patient was turned into prone position onto the standard operating room table. Gel rolls supported the torso thus leaving the abdomen free. Arms were kept up (“Superman position”) and unrestrained. Patients repositioned their arms, upper body, neck and head as needed for comfort throughout the procedure. Pillows, foam headrests or blankets supported the patient’s head, again according to comfort with the option to reposition periodically during the case.

Following the induction of general endotracheal anesthesia, the GA patient was also turned into prone position onto the standard operating room table with the torso supported by rolls. The arms were typically kept up. The head was typically supported with a foam headrest.

For all patients the lumbar spine was prepped and draped, and the decompressive procedure carried out (laminectomy, discectomy or foraminotomy). All procedures were open. A midline spinal incision was created, spanning the level of interest. A portable localizing radiograph was obtained to confirm the spinal level. After removal of all or parts of lamina under direct vision, the remaining dissection and decompression were completed with the aid of an operating microscope. The adequacy of the decompression was assessed with visual inspection and palpation. After obtaining hemostasis, the incision was infiltrated with 10 to 20 ml of 0.5% bupivacaine. It was approximated over a drain using a 3-layer absorbable suture closure.

After dressing application, the patient was turned onto a stretcher in the supine position. GA patients were emerged from anesthesia with reversal of neuromuscular blockade as needed after which the trachea was extubated. SA patients were allowed to sit up as desired for comfort; their subsequent activity was not restricted as long as a headache was not present. SA patients ambulated as desired upon return of motor function.

In the 3 cases where the punctate hole in the dura from the spinal anesthesia injection was visible in the surgical field, the durotomy was repaired by gluing a 3mm piece of muscle to the dura with cyanoacrylate glue.

### Data extraction

With a structured query, predefined data were extracted from records collected by our anesthesia information management system. Measured variables included: 1) demographics (age, ASA physical status classification, gender), 2) anthropometric data (height, weight, and BMI), and 3) type and dose of spinal medication. Measured variables from time of entry into the operating room through time of departure from the operating room included 1) type of vasopressor use (phenylephrine boluses, phenylephrine infusions, ephedrine boluses), 2) type of agents to lower blood pressure (labetolol, metoprolol, esmolol, hydralazine), and 3) total number of intravenous medications administered. The total dose of vasoactive drugs recorded in the AIMS was not included in the data set out of concern for inconsistent manual entry of drug dosing such as changes in the rate of phenylephrine infusions. Entry of the event that a vasoactive agent or some other drug was administered during the case was believed to be reliable.

Drugs administered in the preoperative holding area and drugs administered in the recovery area were not included in the counts because electronic documentation was not employed during the first few years of the data set. Similarly, we did not collect recovery area (PACU) pain scores, nausea/vomiting data, time to readiness for discharge from the PACU or other parameters captured only in handwritten documents for patients from the initial years of the study.

### Power analysis

There was no a priori power calculation for this retrospective study and we collected electronic medical record data based on data availability. However, utilizing a two independent-sample two-tailed t-test assuming power = 0.8 and alpha = 0.05, we were able to detect an effect size as small as d = 0.3 with our observed data (N = 763) and this effect size was considered clinically meaningful.

### Statistical analysis

Descriptive analyses were conducted on both continuous and categorical variables. We report continuous variables using mean and standard deviation or median and quantiles depending on the data distribution. Categorical variables were reported using frequencies and percentages. Appropriate statistical tests (e.g. t-test or Wilcoxon rank sum test for continuous variables, Chi-squared tests or Fisher's exact tests for categorical variables) were selected depending on the variable type and statistical distribution for univariate analyses. The multi-variable primary analysis utilized a restricted maximum likelihood (REML)-based linear mixed effect model approach [29–31]. The dependent variable was the total number of drugs. The analyses included the fixed effects of case procedure, American Society of Anesthesiologist (ASA) class, patient gender, spinal or general anesthesia group, service year, time in hours from OR entry to departure and patient BMI. We used masked physician-identifications (IDs) as random intercepts to build our random intercept mixed effect model.

We conducted an additional practice pattern change analyses to discover the relationship between time (2008–2011 vs. 2012–2016) and the total number drugs administered in different anesthesia types using a two-tailed, two independent sample, Wilcoxon rank sum test.

With propensity score matching (PSM) methods we accounted for potential selection bias introduced by unbalanced assignment to treatment. The propensity score model included age, BMI, ASA, case procedures, service year, OR time and attending categories. The balance of propensity score distribution was examined using histograms and reported in S2 Fig. A two-tailed t-test was conducted to examine the difference of total number of drugs between two study groups in PSM matched sample.

Over one-third of the spinal anesthesia cases were supervised by one particular anesthesiologist (author RAP). To check for potential biasing influence, we conducted a series of sensitivity analyses to compare drug usage (e.g. total number of drugs, ephedrine, phenylephrine usage, etc.) between cases supervised by this anesthesiologist and the cases of all other staff. We applied Chi-squared or Fishers’ exact tests to the data. We also re-conducted the main mixed effect model analyses using a subset of the original sample excluding this particular physician to examine whether our main conclusion changed (See S1 and S2 Tables).

All analyses were performed using R statistical programming software V 3.3.2 and Rstudio V1.0 (Rstudio.inc, Boston, MA). All tests were two-tailed. P-values less than 0.05 were considered as statistically significant.

## Results

A total of 763 consecutive cases were identified for this study, with 619 patients in the GA group and 144 patients in the SA group. Demographic data appear in Table 1. The distribution of surgical procedures was similar between the SA and GA groups (Table 2, Chi-squared test p = 0.717).

**Table 1:** Demographics.

	General Anesthesia	Spinal Anesthesia		
	Median[Q1,Q3]	Median[Q1,Q3]	Median Difference (95% CI)	p value
Age (Years)	65.0[55.0,72.0]	69.5[61.0,76.3]	-5(-7,-3)	<0.001
BMI	26.7[23.5,30.7]	25.2[21.6,28.9]	1.6(0.5,2.9)	0.002
OR Time (min)	176.0[156.0,203.0]	151.5[132.5,170.0]	26(20,32)	<0.001
ASA Class	N (%)	N (%)		0.790*
1	26(4.3%)	5(3.6%)		
2	429(70.1%)	96(68.1%)		
3	155(25.3%)	39(27.7%)		
4	2(0.3%)	1(0.7%)		
Sex	N (%)	N (%)		0.106^#^
F	240(38.8%)	67(46.5%)		
M	379(61.2%)	77(53.5%)		

Patient sex data were available for all patients. The overall study population consisted of 307 women and 456 men. Data for patient age were available for 143 of the 144 SA patients and all GA patients. Sufficient anthropometric data for BMI calculations were incomplete or unavailable for 79 patients in the GA group and 24 patients in the SA group. Data for the ASA status were available for 141 of 144 SA patients and 612 of the 619 GA patients.

#Chi-Squared Test

*Fisher’s Exact Test

**Table 2:** Surgical procedures.

	General Anesthesia	Spinal Anesthesia	
Procedure	N (%)	N (%)	p value
Lumbar Decompression	437(70.6%)	106(73.6%)	0.717^#^
Lumbar Foraminotomy	47(7.6%)	11(7.6%)	
Lumbar Microdiscectomy	135(21.8%)	27(18.8%)	

#Chi-Squared Test

### Total number of drugs administered

**Fig 1:**
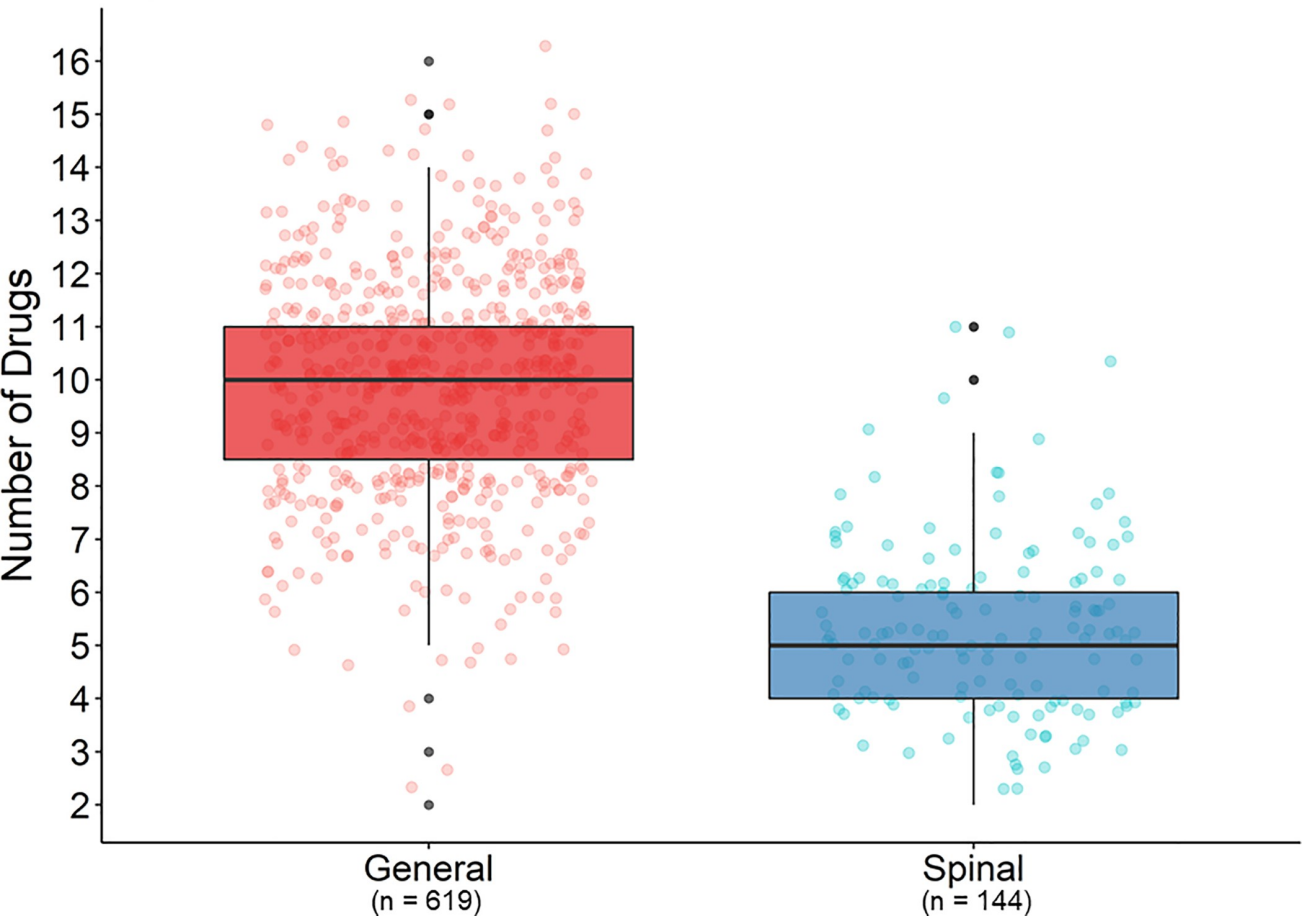
Number of drugs administered by group. Total number of drugs administered for each type of anesthetic. For each group the plot shows all data points, median, Interquartile Range (IQR) and outliers (black circles).

For GA patients, the mean of total number of drugs administered per case was 10 (SD = 2, Q1-Q3: 8–11). For SA patients, the mean of total number of drugs administered per case was 5 (SD = 2, Q1-Q3: 4–6). There was a statistically significant difference between SA and GA groups from the univariate analysis using a linear mixed effects model (difference in mean = -5, 95% CI -5 to -4, p<0.001, Fig 1). In a multivariable mixed model analysis, the difference between SA and GA cases persisted (difference in mean = -4, 95% CI -5 to -4) after controlling for important confounders (e.g. age, gender, BMI, OR time and service year, etc., Table 3). The result therefore substantiates the finding of the univariate analysis showing that SA patients received about half the number of drugs per case than received by GA patients. Our PSM analyses yield similar results; the total number of drugs used in the spinal anesthesia group was 5 drugs less than the general anesthesia group (t-test, 95% CI -5 to -4, P < 0.001). Details appear in S4 Fig.

**Table 3:** Number of drugs administered per case–mixed effect modeling*^.

	Parameter	2.5% CI~	97.5% CI~	p value
Age (yrs.)	0	0	0	0.719
Gender (Male)	-0.4	-0.6	-0.1	0.008
BMI	0	0	0	0.607
OR Time (hr)	0.6	0.1	0.6	0.002
Date of Service (ref = Year 2008)^#^				
2009	-0.1	-0.8	0.6	0.837
2010	1.1	0.4	1.7	0.003
2011	0.9	0.2	1.6	0.015
2012	0.9	0.2	1.6	0.014
2013	1.5	0.8	2.3	<0.001
2014	1.7	1	2.4	<0.001
2015	2.2	1.5	2.9	<0.001
2016	2.4	1.5	3.4	<0.001
ASA Class (ref = ASA class 1)				
ASA Class 2	1.0	0.3	1.7	0.007
ASA Class 3	0.9	0.1	1.6	0.022
ASA Class 4	3.1	0.9	5.4	0.004
Procedure (ref = Lumbar Decompression)				
Lumbar Foraminotomy	0	-1	1	0.632
Lumbar Microdiscectomy	-0.3	-0.7	0	0.060
Spinal Anesthesia (ref = GA)	-4	-5	-4	<0.001

The results show that when adjusting for the indicated factors, the difference between the numbers of drugs administered in SA vs. GA persisted. We failed to detect a statistically significant effect of BMI, age or the procedures on the number of drugs administered. Male gender, high ASA ratings and longer OR time correlate with the administration of a greater number of different drugs. The number of different drugs also increased significantly after 2009 suggesting a practice change over time.

^Some results were reported at one decimal place to avoid bias if rounded. Main results were reported based on the actual measurement precision decimal place (zero decimal place).

* Linear mixed effect model pre-requisites were examined for data distribution.

# Service year was a factor variable and the reference group was year 2008.

~ Variables with 95% confidence intervals not including zero were considered statistically significant.

**Fig 2:**
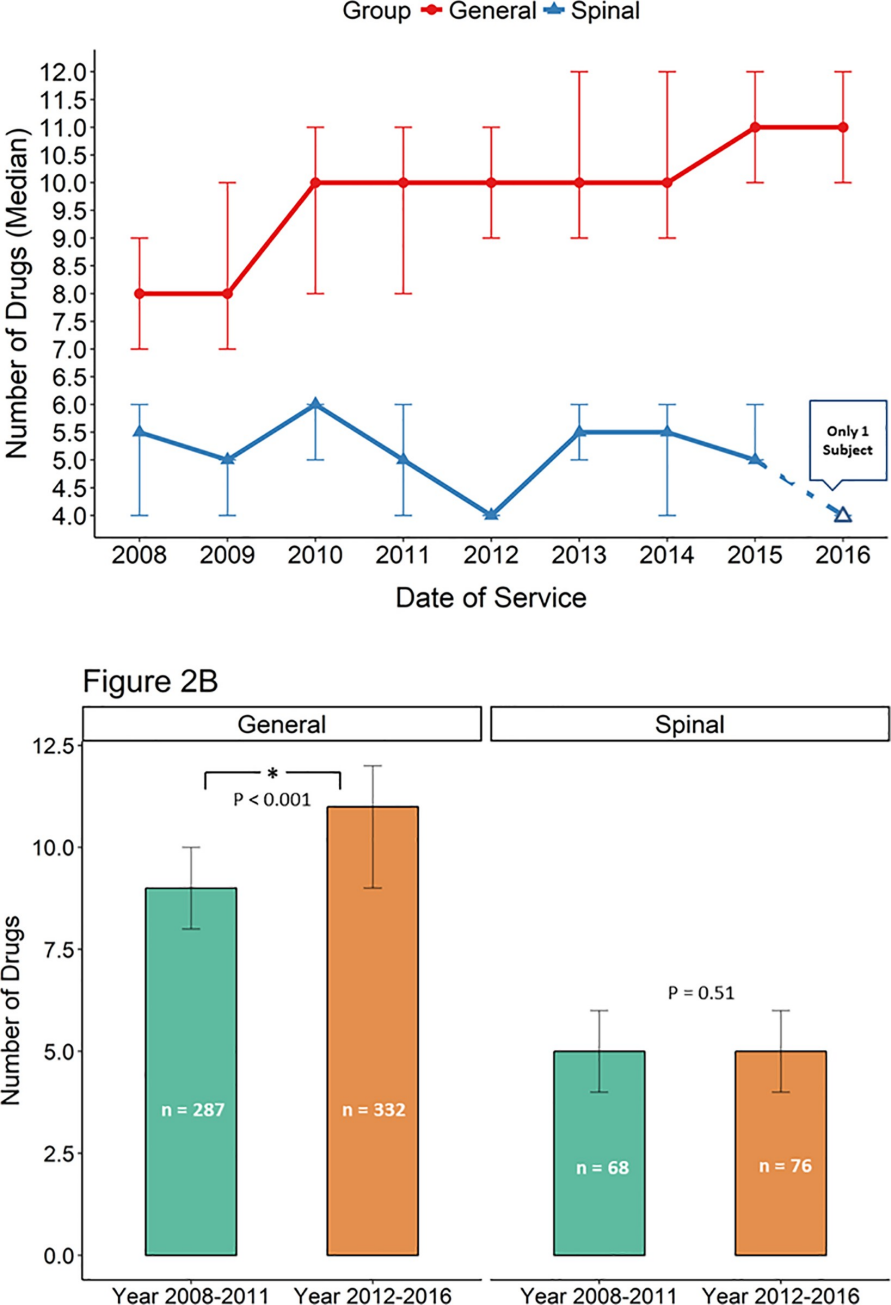
Number of drugs over time. **—**Panel A Number of drugs administered by year (median, quantiles as error bars).—Panel B Number of drugs administered for the first and second halves of each cohort. There was no statistically significant difference between the first half and second half for SA cases. There was a statistically significant difference between the first half and second half GA cases, p <0.001 (Wilcoxon rank sum test). Error bars represent Q1 and Q3.

To assess for possible changes in anesthesia management practice over time, data were subdivided by year (Fig 2: Panel A). Differences between SA and GA appear to be generally consistent over the course of the study suggesting that overall practice did not change. In addition, data from the first half (2008–2011) of each cohort were compared to data from the second half (2012–2016), (Fig 2: Panel B). The total number of drugs significantly increased in the GA group in year 2012–2016 (9 vs. 11, p < 0.001, difference in median = -2, 95% CI: -2 to -1, p<0.001) while the number of drugs remained stable for SA group (5 vs. 5, difference in median = 0, 95% CI: 0 to 1, p = 0.512). Taken together, the data suggest that SA patients received about half the number of drugs received by GA patients.

### Vasoactive agents administered

Vasopressor use frequency (phenylephrine by bolus, phenylephrine by infusion, ephedrine by bolus) was significantly lower in the SA group (Table 4). Similarly, the number of patients receiving a combination of vasopressors was significantly lower in the SA group (Table 4). To control for change in anesthesia hemodynamic management practice over time, data from the first half of each cohort were compared to data from the second half (Table 5). The data for the SA group are similar, suggesting that practice stayed consistent for this cohort (Chi-squared analyses). Interestingly, the frequency of vasopressor use was higher in the later group of GA patients compared to the earlier group of the GA cohort (Chi-squared tests).

**Table 4:** Hemodynamic management: Vasopressor administration spinal vs. general anesthesia: All cases.

		General Anesthesia	Spinal Anesthesia	
Individual Drug Analysis				p value
Phenylephrine				
	Infusion	412 (66.6%)	66 (45.8%)	<0.001
	Bolus	253 (40.9%)	29 (20.1%)	<0.001
Drug Combination Analysis				
Ephedrine	Bolus	219 (35.4%)	23 (16.0%)	<0.001
Phenylephrine	Infusion			
Ephedrine	Bolus	134 (21.7%)	14 (9.7%)	0.002
Phenylephrine	Bolus			
Phenylephrine	Bolus	198 (32.0%)	24 (16.7%)	<0.001
Phenylephrine	Infusion			
Ephedrine	Bolus	111 (17.9%)	12 (8.3%)	0.007
Phenylephrine	Infusion			
Phenylephrine	Bolus			

Treatments were ephedrine by bolus, phenylephrine by bolus, and phenylephrine by infusion. Each case was reviewed for the administration of any of the three treatments (Individual Drug Analysis) and also for combinations of 2 or 3 agents (Drug Combination Analysis). Chi-squared Tests.

**Table 5:** Hemodynamic management: Vasopressor administration spinal vs. general anesthesia: First half vs. second half of each cohort.

		Spinal Anesthesia			General Anesthesia		
		2008–2011	2012–2016		2008–2011	2012–2016	
		N = 68	N = 76		N = 287	N = 332	
		N (%)	N (%)	p value	N (%)	N (%)	p value
Individual Drug Analysis							
Phenylephrine							
	Infusion	35 (51.5)	31 (40.8)	0.264	158 (55.1)	254 (76.5)	<0.001
	Bolus	16 (23.5)	13 (17.1)	0.452	75 (26.1)	178 (53.6)	<0.001
Ephedrine	Bolus	16 (23.5)	22 (28.9)	0.584	126 (43.9)	177 (53.3)	0.024
Drug Combination Analysis							
Ephedrine	Bolus	12 (17.6)	11 (14.5)	0.771	75 (26.1)	144 (43.4)	<0.001
Phenylephrine	Infusion						
Ephedrine	Bolus	7 (10.3)	7 (9.2)	1	35 (12.2)	99 (29.8)	<0.001
Phenylephrine	Bolus						
Phenylephrine	Bolus	14 (20.6)	10 (13.2)	0.332	52 (18.1)	146 (44.0)	<0.001
Phenylephrine	Infusion						
Ephedrine	Bolus	7 (10.3)	5 (6.6)	0.615	24 (8.4)	87 (26.2)	<0.001
Phenylephrine	Infusion						
Phenylephrine	Bolus						

Treatments were ephedrine by bolus, phenylephrine by bolus, and phenylephrine by infusion. Each case was reviewed for the administration of any of the three treatments (Individual Drug Analysis) and also for combinations of 2 or 3 agents (Drug Combination Analysis). Chi-squared Tests.

Review of the anesthesia records identified four vasoactive agents to reduce blood pressure and/or heart rate: esmolol, metoprolol, hydralazine and labetolol. Esmolol was administered to 51 of 619 GA patients (8.24%) but only 2 of 144 SA patients (1.39%), p = 0.002 by Fisher’s Exact test. Labetolol was administered to 58 of 619 GA patients (9.37%) and 3 of 144 SA patients (2.08%), p = 0.002 by Fisher’s Exact test. Ten GA patients received both esmolol and labetolol. The proportions of patients receiving metoprolol (GA 1.94%, SA 0.69%, p = 0.239) or hydralazine (GA 0.48%, SA 1.39%, p = 0.480) did not differ, p>0.05, but the number of administrations was very small.

### Spinal anesthesia drugs and success rate

**Fig 3:**
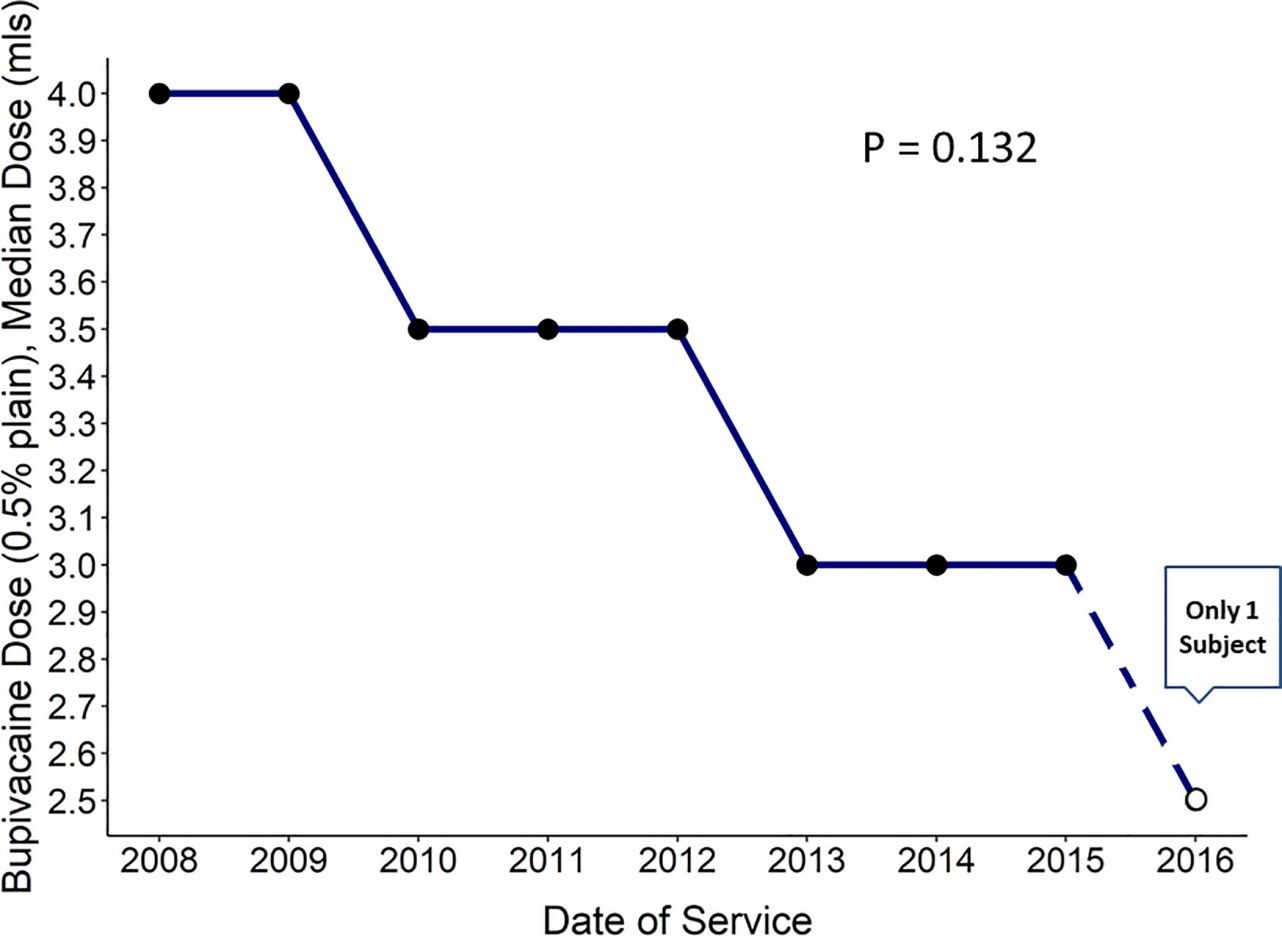
Bupivacaine spinal anesthetic dose. Bupivacaine, 0.5% solution without any adjuvant agents. Median dose (milliliters) by year. The apparent downward trend in dose over time was not statistically significant (p = 0.132, linear regression).

For the SA group, the spinal anesthetic medication and dosing were unavailable for 2 patients leaving a total of 142 patients to be analyzed for this data item. Isobaric bupivacaine (0.5% (w/v)) with no adjuvants was used for 134 patients with an average dose of 3.32 mL (range 2.5–5.0 mL [12.5–25.0 mg]). Inspection of a dose vs. time plot suggests that the dose of isobaric bupivacaine decreased over time; this apparent reduction was not statistically significant (Fig 3, p = 0.132 from linear regression). Three patients received hyperbaric 0.75% (w/v) bupivacaine, with 2 patients receiving doses documented as being 2 ml (15 mg). The third patient received hyperbaric bupivacaine, 1.6 ml (12 mg). Four patients received bupivacaine, 0.5%, with epinephrine (1:200, 000), 3 ml. One patient received isobaric bupivacaine, 0.25%, 6 ml.

Of the 144 cases intended to be managed by SA, one case was canceled after successful placement of the spinal anesthetic when the patient experienced respiratory distress following prone positioning. In a second case, the dermatome level necessary for surgery was not achieved and GA was administered to allow surgery to proceed. The presumptive cause was injection of the spinal drug caudal to a tight lumbar stenosis. In 2 other cases the SA block became inadequate during surgery; a laryngeal mask airway was inserted without incident following an induction dose of propofol in both cases. No neurologic complications were attributable to SA or were concealed by SA.

### Sensitivity analysis

The senior author (RAP) supervised the anesthesia management of 55 of the 144 SA cases (38% of cases) and supervised or personally administered the anesthetics for 53 of the 619 cases managed with GA (8.6%). Sensitivity analysis revealed no selection bias towards the main overall conclusions of the study introduced by this single provider (S1–S3 Tables).

## Discussion

### Main findings

In our study population patients undergoing routine lumbar spine surgery receive approximately half the number of intravenous drugs under SA compared to GA. Of the ~5 drugs administered for SA cases, one was the antibiotic and another was the intrathecal agent. Although it is intuitive that fewer drugs would be administered during SA, no previous descriptions of SA for lumbar surgery report such quantitative data. Vasoactive drug administration in SA was less frequent than with GA; we infer that SA confers hemodynamic stability compared to GA. Previous publications vary in their hemodynamic findings but provide fewer hemodynamic management details. It is unclear from some reports whether apparent greater stability with SA or with GA reflects specific intervention, thereby confounding interpretation [6, 9, 10, 12, 17]. Our findings about the frequency of vasopressor use are consistent with a previous report [21].

Overall, these novel findings support our hypothesis that quantitative review of case data would confirm clinical impressions that SA compared to GA is associated with the administration of fewer drugs, including agents to control hemodynamics. If one or more inhaled anesthetic agents is added to the count of GA drugs, the difference between SA and GA in the total number of drugs per anesthetic is even more pronounced.

The data also indicate that successful spinal anesthetics for the selected surgeries can typically be accomplished with bupivacaine, ~3mL, 0.5% isobaric solution (15 mg) confirming other studies [8, 11–13, 19–21]. Adjuvants (epinephrine, opiates) are not necessarily required. Conversion from SA to GA was infrequent.

Selective use of SA confers several advantages as summarized by recent reviews [22–25]. We identify another advantage, a reduction in the number of different drugs used per case. Prospective studies suggested ranges of anesthesia drug administration error/near miss rates [32–34]. Assuming an average error rate of 5% per drug administration [34], and ignoring drug interactions and combinations, a difference of 4–5 in the total number of drugs might suggest a 20–25% increase in drug administration error rate with general anesthesia. With a more conservative estimate of error rate, ~1%, then the increase in error rate is 4–5%. Reducing medication numbers may lessen wrong dose or wrong medication errors, an error prevention strategy [35].

Decreasing the number of administered medications also has implications for perioperative infection control, for which anesthesiologists bear some responsibility [36–38] because they inject medications into stopcocks or ports [39]. Perhaps risks of introducing infection will be lower with SA compared to GA. In this retrospective study of patients undergoing lumbar spine surgery it was not possible to determine the incidence of infection, a measurement that might be attempted in a large prospective trial. Interestingly, incidences of infection in arthroplasty patients were lower with neuraxial anesthesia versus general anesthesia [40, 41].

One concern with SA is hypotension resulting from sympathectomy exacerbated by prone positioning which decreases venous return and preload. Several groups report effects of SA on hemodynamic parameters for patients undergoing lumbar spine surgery. Unfortunately, the exact method to measure blood pressure (BP) and pulse rate (P), and compute changes from baseline, is not always described [4, 11, 20, 27]. Jellish et al found more frequent use of ephedrine in SA cases (36.1% vs 22.9%) although the difference was not statistically significant. Hypertension and tachycardia were more commonly seen in their GA cases, with similar incidence of hypotension. They used as treatment criteria changes of BP and P from baseline +/- 20%, but how the “baseline” was determined is not clearly defined [2]. Sadrolsadat et al compared SA with GA for disc herniation laminectomy. They observed an increased incidence of hypotension in SA patients, concluding that GA provides greater hemodynamic stability [8]. In the study by Pierce et al, vasopressor use was more frequent with SA [17]. Interestingly, our results show less frequent vasopressor use during SA cases which is consistent with a previous finding [21]. We found that cases in our series mainly used isobaric bupivacaine (0.5%) without any adjuvant. In contrast, hyperbaric bupivacaine solutions with or without adjuvants and even tetracaine were employed in other series [2, 9, 10, 17]. Perhaps the choice of spinal drug, and the dose administered, can account for the divergent hemodynamic findings [26].

Other specific concerns with spinal anesthesia include the possibility of nerve injury resulting from the spinal anesthetic or initial masking of injury caused by the surgical procedure. No neurologic complications were attributable to SA or were concealed by SA in our cohort.

### Limitations of the study

This is a retrospective study of consecutive patients and is thus not randomized, unlike others report [2, 8–11, 18, 20]. There is potential for unbalanced groups, although our propensity matching and multivariable analyses help account for sources of bias. Is a truly randomized study with consecutive patients feasible, with results that can be generalized to real world practice? Patient choice potentially confounds efforts to conduct randomized studies aimed at comparing SA to GA. Patients may prefer GA because of factors such as anxiety which precludes obtaining consent to participate in a randomized study.

Anesthesiologists must also consider potential intraoperative SA failure. Airway management when converting to GA is most expeditiously addressed by inserting an LMA, which is possible and safe for many, but not all, prone patients. If judgment and experience raise concerns about prone airway management, a randomized assignment to SA may be unwise. Is the individual patient’s body habitus conducive to comfort, particularly respiratory comfort, when positioned prone? This determination is also based on judgment and experience. Will the level of stenosis interfere with the rostral spread of a spinal drug to a level necessary to anesthetize the site of surgery? The surgeon’s assessments factor into the anesthesia choice as well, based on estimates of surgical complexity and duration. Consequently, a truly randomized prospective study of consecutive patients comparing SA to GA for lumbar spine surgery may not be feasible. The conclusions from a study enrolling only a select patient subset may be difficult to generalize for the real world.

This retrospective study has other limitations. During the early years of the study, data on co-morbidities were recorded on inaccessible paper documents or in unstructured electronic notes that could not be searched. Patients come exclusively from one surgeon’s practice, although other studies have the same feature [16–18]. Patients were positioned on a standard table, supported by rolls; SA patients might be uncomfortable on other tables. We lack complete data for several records, although overall data capture rates were high. Anesthetics were managed at the team’s discretion, not according to protocol, potentially introducing variability in the results. Inferences about hemodynamics derive from data on the incidence of pressor administration. We lack clinical data, such as the need for postoperative analgesics, beyond the operating room, because this information was recorded on handwritten, unstructured, paper records for the majority of the study interval.

We did not extract BP and P data, another limitation of this study. Hemodynamic fluctuations, and their duration, at induction, intubation, emergence and extubation associated with GA, and hemodynamic fluctuations with change of position from supine to prone and then prone to supine in both SA and GA, are difficult to coalesce for quantitative comparisons to baseline hemodynamics. What constitutes individual baseline BP for such comparisons? Different sources of “baseline”BP, e.g. preoperative vs. pre-induction, can confound establishing reference values for perioperative BP management [42, 43]. Absolute blood pressure criteria for intervention (e.g. P < 60 bpm or SBP < 90mmHg) [9, 10, 44] may not account for individual differences in baseline hemodynamics.

If precisely defining intraoperative hypotension relative to baseline can be challenging [45], an advantage of our study is that no specific protocols or criteria guided BP management. Data on the incidence of vasoactive drug administration reflect clinical assessments and management decisions for individual patients. For the SA cohort there were 38 different staff anesthesiologists; 115 individual members of the anesthesia staff managed GA cases. Therefore, our data provide a window into real world anesthesia care.

## Conclusions

Our findings confirm and extend the observations of other investigators. For selected patients, for specific lumbar spine surgeries, SA may be a reasonable option. Possible safety advantages include reduced number of different drugs administered during SA, thereby lessening potentials for medication error, adverse drug-drug interactions and postoperative infections attributable to administering intravenous medications. Other advantages include apparent hemodynamic stability and reduced OR time. Results appear to be consistent despite the number of independent providers and the absence of clinical protocols, suggesting that the observations reflect real world practice.

## Supporting information

S1 Fig Lumbar spine pathology influences the choice of GA vs. SA. Panel A.
An elderly patient presented with gait disturbance, leg pain and neurogenic claudication and found to have lumbar spine stenosis at L4-L5 (arrow head, transitional sacral anatomy). The surgical plan was for L4-L5 decompression. The airway was reassuring. The patient was offered spinal anesthesia which was accomplished uneventfully with insertion of the spinal needle at the estimated L2-L3 interspace and injection of bupivacaine, 0.5%, 2.5 ml resulting in a T6 sensory level. The patient received a total of 5 anesthesia administered drugs which included the spinal drug, the antibiotic, fentanyl (100 mcg at the beginning of the case), an infusion of phenylephrine (documented as 10–30 mcg/minute) and an infusion of propofol (documented as 30–50 mcg/kg/minute).
Panel B.
An elderly patient presented with neurogenic claudication and leg pain and was found to have lumbar spine stenosis at L2-L3, L3-L4 and L4-L5. The patient was not offered the option of spinal anesthesia. Successful spinal anesthesia would have required injection above L2-L3 to achieve adequate rostral spread, with risk of injury to the conus medullaris (arrow). Moreover, the likely complexity of the surgery made case duration unpredictable. The patient received a total of 10 anesthesia-administered intravenous drugs, in addition to the inhalation agent (sevoflurane).
(JPG)

S2 Fig Evaluation of balance of propensity scores for PSM. With propensity score matching (PSM) methods we accounted for potential selection bias introduced by unbalanced assignment to treatment. The propensity score model included age, BMI, ASA, case procedures, service year, OR time and attending categories. The balance of propensity score distribution was examined using histograms and reported in this supplement, This figure shows overlapping areas representing the PS score distribution between General anesthesia and Spinal anesthesia groups, thereby justifying our choice of PSM for sensitivity analysis.
A two-tailed t-test was conducted to examine the difference of total number of drugs between two study groups in PSM matched samples.
(JPG)

S3 Fig PSM analysis code. (JPG)

S4 Fig Random effects. Random intercepts for each attending from the random intercept mixed model. The outlined gray point represents provider RAP. Black points represent other physicians. The size of the point indicates the number of cases conducted by each provider.
This random effects plot shows that all points are organized between -0.5 to +0.5 around the central line of 0.0. Author RAP had a tendency to administer fewer drugs in his practice compared with the grand mean of the number of drugs administered by all other anesthesia attendings (intercept less than zero). However, these practice differences were relatively small, suggesting the effects of provider variability are properly addressed by the implemented random effects model.
(JPG)

S1 Table Number of drugs administered per case: Sensitivity analysis senior author RAP vs. ALL OTHER attending staff anesthesiologists. Two independent-sample two-tailed Wilcoxon rank sum test for both GA and SA patients. There was no statistical significant difference between RAP and other physician in terms of number of drugs used in GA group (p = 0.342). However, there was a statistical significant difference between RAP and other physicians in SA group (p = 0.004). Our results indicated that RAP might have a different practice pattern compared with other physicians (less drugs used) for SA.
GA: General Anesthesia.
SA: Spinal Anesthesia.
(DOCX)

S2 Table Number of drugs administered per case: ALL OTHER attending staff anesthesiologists–mixed effect modeling*^. The results show that after removing cases staffed by senior author RAP, and when adjusting for the indicated factors, the difference between the numbers of drugs administered in SA vs. GA persists. Similar to the analysis of the entire cohort (Table 3), there was no statistically significant effect of BMI, or the procedure on the number of drugs. Male gender, high ASA ratings and longer OR time continue to correlate with the number of drugs used. The significant increase in the number of drug used over time also remains.
^Some results were reported at one decimal place to avoid bias if rounded. Main results were reported based on the actual measurement precision decimal place (zero decimal place).
* Linear mixed effect model pre-requisites were examined for data distribution.
# Service year was a factor variable and the reference group was year 2008.
~ Variables with 95% confidence intervals not including zero were considered statistically significant.
(DOCX)

S3 Table Hemodynamic management: Vasopressor administration spinal vs. general anesthesia: Sensitivity analysis, Senior author RAP vs. ALL OTHER attending staff anesthesiologists. Hemodynamic management was compared between senior author RAP and ALL OTHER attending anesthesiologists. We conducted Chi-squared tests to compare drug usages between RAP and other attendings. A p value less than 0.05 was considered statistically significant and no post-hoc adjustments applied in this analysis. There was no statistical significant difference between RAP and other attendings for drugs used in hemodynamic management.
(DOCX)

S1 Data De-identified study data. (ZIP)
