# Supplementary figures and images for: Spinal anesthesia for lumbar spine surgery correlates with fewer total medications and less frequent use of vasoactive agents: A single center experience
Source: PLoS One. 2019 Jun 13;14(6):e0217939. doi: 10.1371/journal.pone.0217939 (PMC6563985; doi:10.1371/journal.pone.0217939)

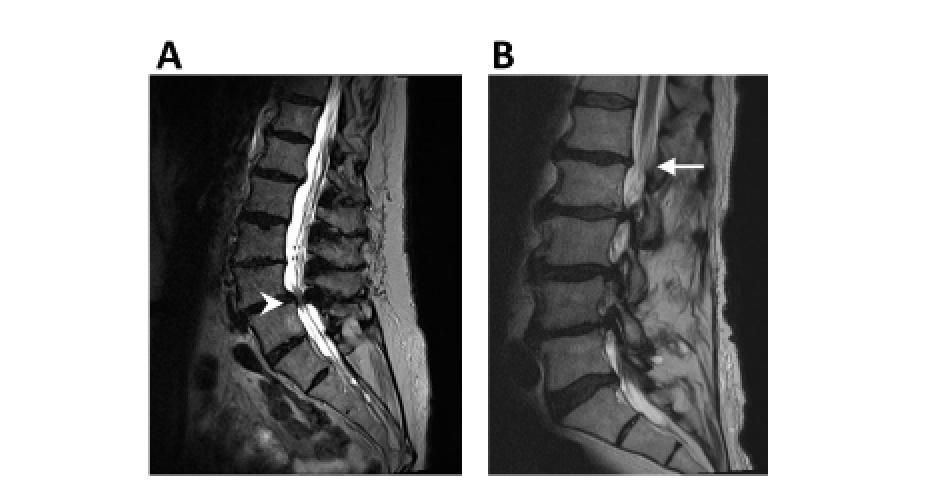

Supplement: S1 Fig — Panel A. An elderly patient presented with gait disturbance, leg pain and neurogenic claudication and found to have lumbar spine stenosis at L4-L5 (arrow head, transitional sacral anatomy). The surgical plan was for L4-L5 decompression. The airway was reassuring. The patient was offered spinal anesthesia which was accomplished uneventfully with insertion of the spinal needle at the estimated L2-L3 interspace and injection of bupivacaine, 0.5%, 2.5 ml resulting in a T6 sensory level. The patient received a total of 5 anesthesia administered drugs which included the spinal drug, the antibiotic, fentanyl (100 mcg at the beginning of the case), an infusion of phenylephrine (documented as 10–30 mcg/minute) and an infusion of propofol (documented as 30–50 mcg/kg/minute). Panel B. An elderly patient presented with neurogenic claudication and leg pain and was found to have lumbar spine stenosis at L2-L3, L3-L4 and L4-L5. The patient was not offered the option of spinal anesthesia. Successful spinal anesthesia would have required injection above L2-L3 to achieve adequate rostral spread, with risk of injury to the conus medullaris (arrow). Moreover, the likely complexity of the surgery made case duration unpredictable. The patient received a total of 10 anesthesia-administered intravenous drugs, in addition to the inhalation agent (sevoflurane). (JPG) [file pone.0217939.s001.jpg]

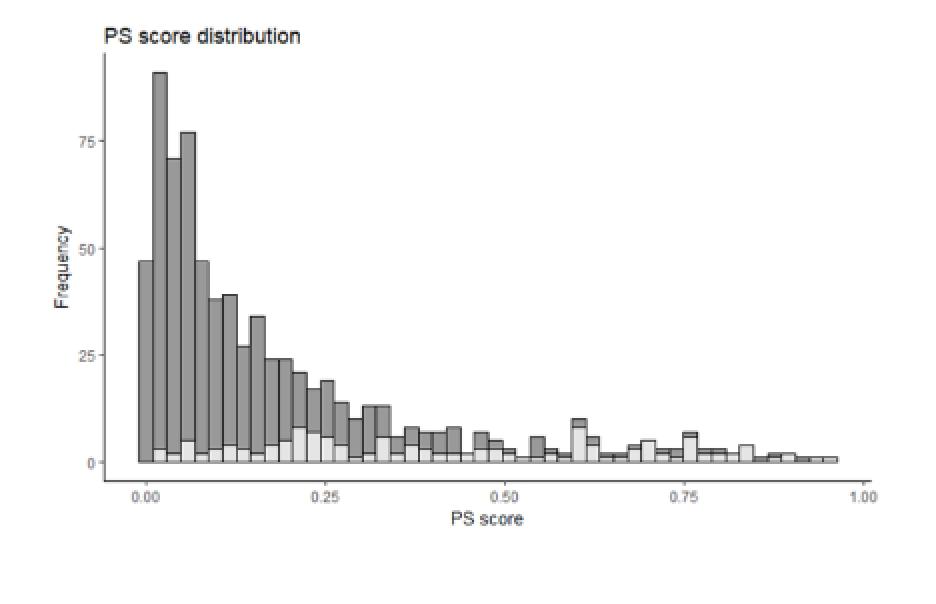

Supplement: S2 Fig — With propensity score matching (PSM) methods we accounted for potential selection bias introduced by unbalanced assignment to treatment. The propensity score model included age, BMI, ASA, case procedures, service year, OR time and attending categories. The balance of propensity score distribution was examined using histograms and reported in this supplement, This figure shows overlapping areas representing the PS score distribution between General anesthesia and Spinal anesthesia groups, thereby justifying our choice of PSM for sensitivity analysis. A two-tailed t-test was conducted to examine the difference of total number of drugs between two study groups in PSM matched samples. (JPG) [file pone.0217939.s002.jpg]

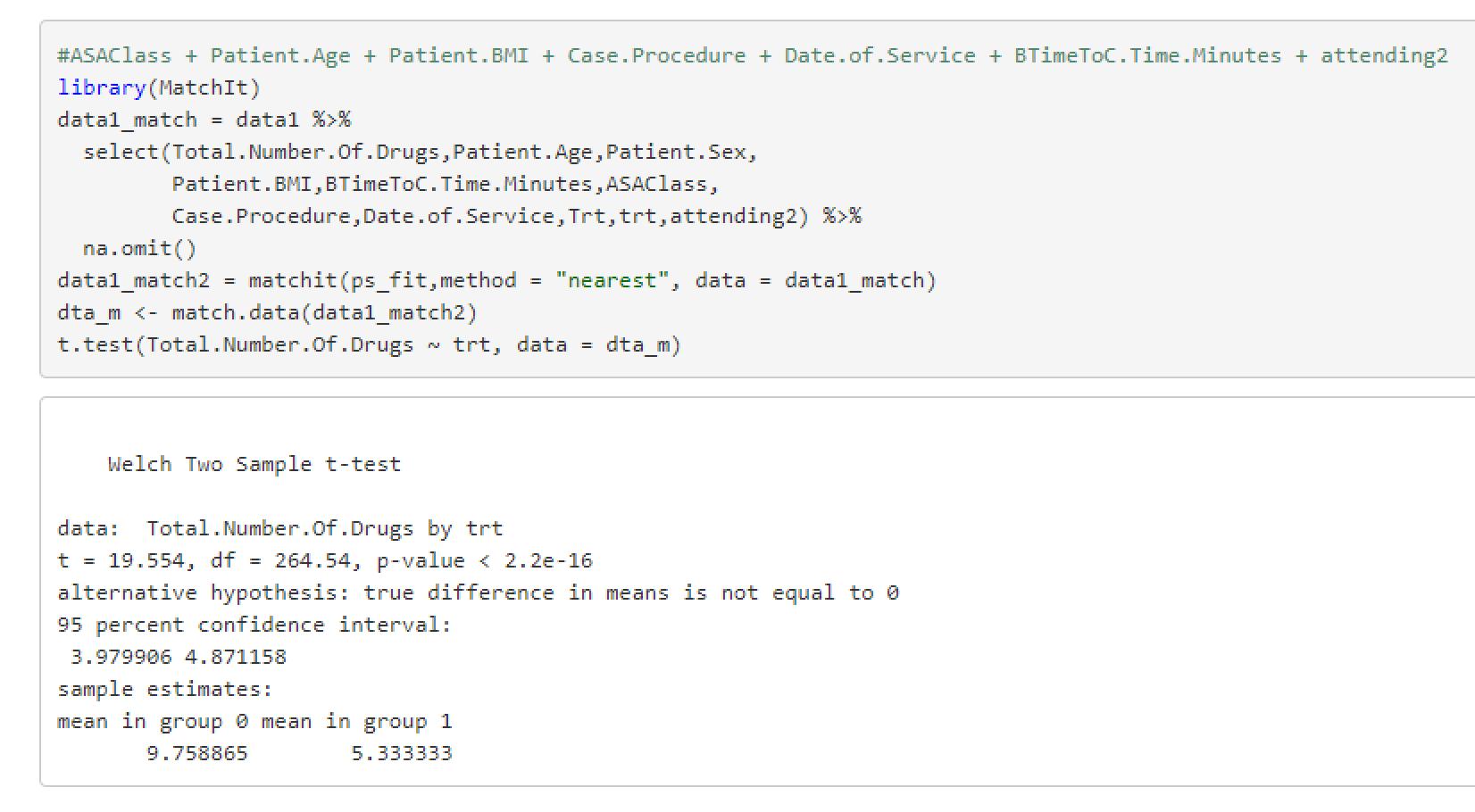

Supplement: S3 Fig — (JPG) [file pone.0217939.s003.jpg]

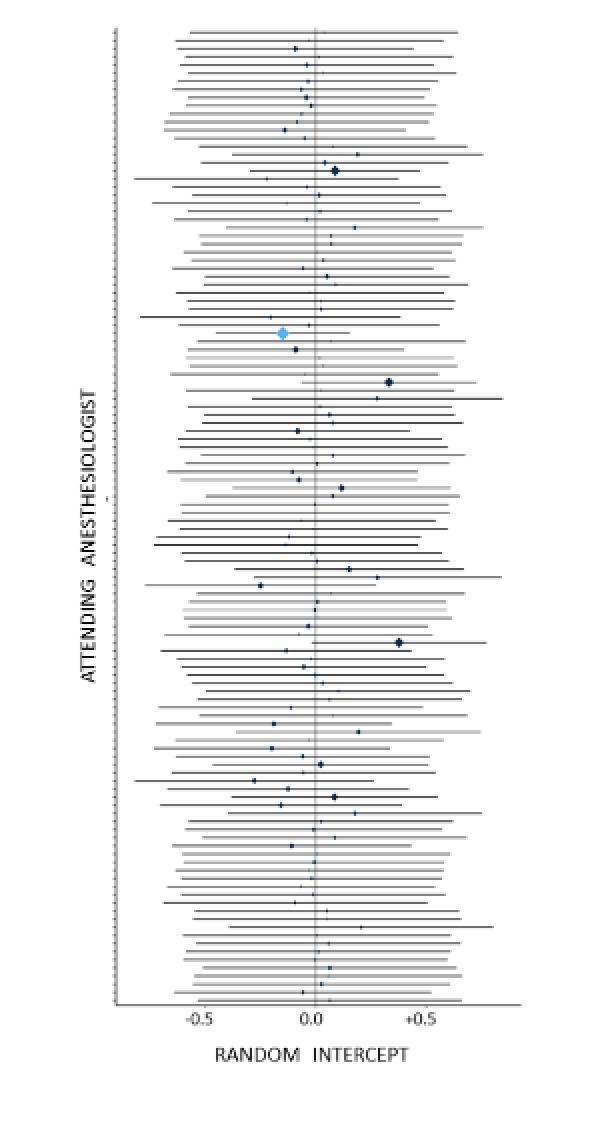

Supplement: S4 Fig — Random intercepts for each attending from the random intercept mixed model. The outlined gray point represents provider RAP. Black points represent other physicians. The size of the point indicates the number of cases conducted by each provider. This random effects plot shows that all points are organized between -0.5 to +0.5 around the central line of 0.0. Author RAP had a tendency to administer fewer drugs in his practice compared with the grand mean of the number of drugs administered by all other anesthesia attendings (intercept less than zero). However, these practice differences were relatively small, suggesting the effects of provider variability are properly addressed by the implemented random effects model. (JPG) [file pone.0217939.s004.jpg]
