# Supplementary material for: Spinal anesthesia for lumbar spine surgery correlates with fewer total medications and less frequent use of vasoactive agents: A single center experience
Source: PLoS One. 2019 Jun 13;14(6):e0217939. doi: 10.1371/journal.pone.0217939 (PMC6563985; doi:10.1371/journal.pone.0217939)
Supplement: S1 Table — Two independent-sample two-tailed Wilcoxon rank sum test for both GA and SA patients. There was no statistical significant difference between RAP and other physician in terms of number of drugs used in GA group (p = 0.342). However, there was a statistical significant difference between RAP and other physicians in SA group (p = 0.004). Our results indicated that RAP might have a different practice pattern compared with other physicians (less drugs used) for SA. GA: General Anesthesia. SA: Spinal Anesthesia. (DOCX) [file pone.0217939.s005.docx]

|  | RAP | ALL OTHERS | Median Difference | 95% CI | p value |
| --- | --- | --- | --- | --- | --- |
| **GA** | 10 [9, 12] | 10 [8, 11] | 0 | 0 to 1.0 | 0.342 |
| **SA** | 5 [4, 6] | 6 [4, 7] | -1 | -1.0 to 0 | 0.004 |
