# Supplementary material for: Spinal anesthesia for lumbar spine surgery correlates with fewer total medications and less frequent use of vasoactive agents: A single center experience
Source: PLoS One. 2019 Jun 13;14(6):e0217939. doi: 10.1371/journal.pone.0217939 (PMC6563985; doi:10.1371/journal.pone.0217939)
Supplement: S2 Table — The results show that after removing cases staffed by senior author RAP, and when adjusting for the indicated factors, the difference between the numbers of drugs administered in SA vs. GA persists. Similar to the analysis of the entire cohort (Table 3), there was no statistically significant effect of BMI, or the procedure on the number of drugs. Male gender, high ASA ratings and longer OR time continue to correlate with the number of drugs used. The significant increase in the number of drug used over time also remains. ^Some results were reported at one decimal place to avoid bias if rounded. Main results were reported based on the actual measurement precision decimal place (zero decimal place). * Linear mixed effect model pre-requisites were examined for data distribution. # Service year was a factor variable and the reference group was year 2008. ~ Variables with 95% confidence intervals not including zero were considered statistically significant. (DOCX) [file pone.0217939.s006.docx]

|  | Parameter | 2.5% CI ~ | 97.5% CI~ | p value |
| --- | --- | --- | --- | --- |
| Age (yrs.) | 0.0 | 0.0 | 0.0 | 0.973 |
| Gender (Male) | -0.4 | -0.7 | -0.1 | 0.007 |
| BMI | 0.0 | 0.0 | 0.0 | 0.845 |
| OR Time (hr) | 0.3 | 0.1 | 0.6 | 0.007 |
| Date of Service (ref = Year 2008)^#^ |  |  |  |  |
| 2009 | -0.1 | -0.9 | 0.6 | 0.724 |
| 2010 | 1.2 | 0.4 | 2.0 | 0.002 |
| 2011 | 1.0 | 0.2 | 1.7 | 0.016 |
| 2012 | 1.0 | 0.2 | 1.8 | 0.015 |
| 2013 | 1.7 | 0.8 | 2.5 | <0.001 |
| 2014 | 1.9 | 1.1 | 2.7 | <0.001 |
| 2015 | 2.3 | 1.5 | 3.1 | <0.001 |
| 2016 | 2.6 | 1.6 | 3.7 | <0.001 |
| ASA Class (ref = ASA Class 1) |  |  |  |  |
| ASA Class 2 | 1.0 | 0.3 | 1.8 | 0.008 |
| ASA Class 3 | 0.9 | 0.0 | 1.7 | 0.039 |
| ASA Class 4 | 3.3 | 1.0 | 5.5 | 0.005 |
| Procedure (ref = Lumbar Decompression) |  |  |  |  |
| LUMBAR FORAMINOTOMY | 0.4 | -0.2 | 0.9 | 0.185 |
| LUMBAR MICRODISCECTOMY | -0.4 | -0.8 | 0.0 | 0.049 |
| Spinal Anesthesia (ref = GA) | -4 | -5 | -4 | <0.001 |
