# Supplementary material for: Spinal anesthesia for lumbar spine surgery correlates with fewer total medications and less frequent use of vasoactive agents: A single center experience
Source: PLoS One. 2019 Jun 13;14(6):e0217939. doi: 10.1371/journal.pone.0217939 (PMC6563985; doi:10.1371/journal.pone.0217939)
Supplement: S3 Table — Hemodynamic management was compared between senior author RAP and ALL OTHER attending anesthesiologists. We conducted Chi-squared tests to compare drug usages between RAP and other attendings. A p value less than 0.05 was considered statistically significant and no post-hoc adjustments applied in this analysis. There was no statistical significant difference between RAP and other attendings for drugs used in hemodynamic management. (DOCX) [file pone.0217939.s007.docx]

|  |  | **GA** | **SA** |
| --- | --- | --- | --- |
|  |  | p value | p value |
| Inidvidual Drug Analysis | |  |  |
| Phenylephrine |  |  |  |
|  | Infusion | 1 | 0.430 |
|  | Bolus | 0.962 | 1 |
| Ephedrine | Bolus | 1 | 0.701 |
| Drug Combination Analysis | |  |  |
| Ephedrine | Bolus | 0.599 | 0.738 |
| Phenylephrine | Infusion |  |  |
| Ephedrine | Bolus | 0.734 | 0.930 |
| Phenylephrine | Bolus |  |  |
| Phenylephrine | Bolus | 0.889 | 1 |
| Phenylephrine | Infusion |  |  |
| Ephedrine | Bolus | 0.999 | 1 |
| Phenylephrine | Infusion |  |  |
| Phenylephrine | Bolus |  |  |
